# Identification of a deep-branching thermophilic clade sheds light on early bacterial evolution

**DOI:** 10.1038/s41467-023-39960-x

**Published:** 2023-07-19

**Authors:** Hao Leng, Yinzhao Wang, Weishu Zhao, Stefan M. Sievert, Xiang Xiao

**Affiliations:** 1 State Key Laboratory of Microbial Metabolism, School of Life Sciences and Biotechnology, Shanghai Jiao Tong University, Shanghai, China; 2 International Center for Deep Life Investigation (IC-DLI), Shanghai Jiao Tong University, Shanghai, China; 3 Biology Department, Woods Hole Oceanographic Institution, Woods Hole, MA USA; 4 Southern Marine Science and Engineering Guangdong Laboratory (Zhuhai), Zhuhai, Guangdong China

**Keywords:** Bacterial evolution, Marine microbiology, Bacterial physiology

## Abstract

It has been proposed that early bacteria, or even the last universal common ancestor of all cells, were thermophilic. However, research on the origin and evolution of thermophily is hampered by the difficulties associated with the isolation of deep-branching thermophilic microorganisms in pure culture. Here, we isolate a deep-branching thermophilic bacterium from a deep-sea hydrothermal vent, using a two-step cultivation strategy (“Subtraction-Suboptimal”, StS) designed to isolate rare organisms. The bacterium, which we name *Zhurongbacter thermophilus* 3DAC, is a sulfur-reducing heterotroph that is phylogenetically related to Coprothermobacterota and other thermophilic bacterial groups, forming a clade that seems to represent a major, early-diverging bacterial lineage. The ancestor of this clade might be a thermophilic, strictly anaerobic, motile, hydrogen-dependent, and mixotrophic bacterium. Thus, our study provides insights into the early evolution of thermophilic bacteria.

## Abstract (web summary)

Thermophilic microorganisms can live at high temperatures, but the origin and evolution of this ability are unclear. Here, the authors isolate a thermophilic bacterium from a deep-sea hydrothermal vent, and show it belongs to a major early-diverging lineage whose ancestor was likely also a thermophilic bacterium.

## Introduction

Organisms with optimum growth temperatures higher than 45 °C are designated as thermophiles^1,2^. These thermophiles are ubiquitously distributed in natural or artificial high-temperature environments, such as terrestrial volcanic sites, hot springs, submarine hydrothermal systems, deep subseafloor, oil reservoirs, solar-heated surface soils and industrial incubators^3–8^. Some thermophiles can also live in alkaline, acidic, or high salinity conditions, highlighting their ability to tolerate multiple stressors^9,10^. These so-called “extreme environments” are generally considered analogs of conditions existing on early Earth when life originated in the late Hadean or early Archaean eon^11,12^, and multiple experimental studies^13,14^ and geological evidence^15^ supported the early origin of thermophiles.

Thermophilic bacteria can be found in a wide range of bacterial phyla, yet only a few phyla contain almost exclusively thermophilic bacteria, namely, Aquificota, Dictyoglomota, Coprothermobacterota, Thermotogota, and Thermodesulfobiota. While it has been suggested that thermophily might represent an ancestral feature of bacteria or even life^16–20^, other hypotheses have put forward a non-thermophilic origin of bacteria and placed bacterial lineages such as Chloroflexota or the Candidate Phyla Radiation (CPR) near the root of the bacterial phylogenomic tree^21–23^. A recent study using an outgroup-free gene tree-species tree reconciliation approach rejected previously predicted bacterial roots and placed a new robust root between two major bacterial branches, i.e., Gracilicutes and Terrabacteria, even though with uncertainties regarding the phylogenetic position of the Fusobacteriota lineage^24^. Nevertheless, it remains unclear when the thermophilic bacteria originated and how their metabolisms evolved.

The predicted metabolic potential of the last bacterial common ancestor (LBCA) or early diverging bacterial lineage can shed light on the early Earth environments and early ecosystem^20,25–28^. For thermophilic bacteria, several previous studies focusing on different lineages such as Thermotogota^29^, Aquificota^30^, and Coprothermobacterota^31^ revealed a complex evolutionary history with a high proportion of genes horizontally transferred from other prokaryotes. However, there is still a lack of an evolutionary study based on comprehensive comparative genomic analyses of major thermophilic bacterial clades. Even though cultivation-free metagenomic analyses have greatly expanded our knowledge of bacterial diversity^32,33^, research on thermophilic bacteria still requires isolated strains to provide direct evidence of their physiology, such as optimal growth temperature to confirm their thermophilic features. Yet, it is extremely difficult to obtain pure cultured novel thermophilic bacteria, especially for deep-branching clades.

Here, we report the discovery and isolation of a novel thermophilic, piezophilic, sulfur-reducing bacterial strain, *Zhurongbacter thermophilus* 3DAC. Phylogenomic studies of the strain 3DAC indicate that it is closely related to major thermophilic bacterial groups, such as Coprothermobacterota, Dictyoglomota, Caldisericota, and Thermotogota. Phylogenetic ancestral analyses indicate that these closely clustered thermophilic bacterial lineages share one last common ancestor with the potential to metabolize hydrogen, sulfur compounds, and with mixotrophic carbon catabolism.

## Results and discussion

### Discovery and isolation of a previously unknown thermophilic and piezophilic bacterium

#### Isolation with a novel “Subtraction-Suboptimal” strategy

**Fig. 1:**
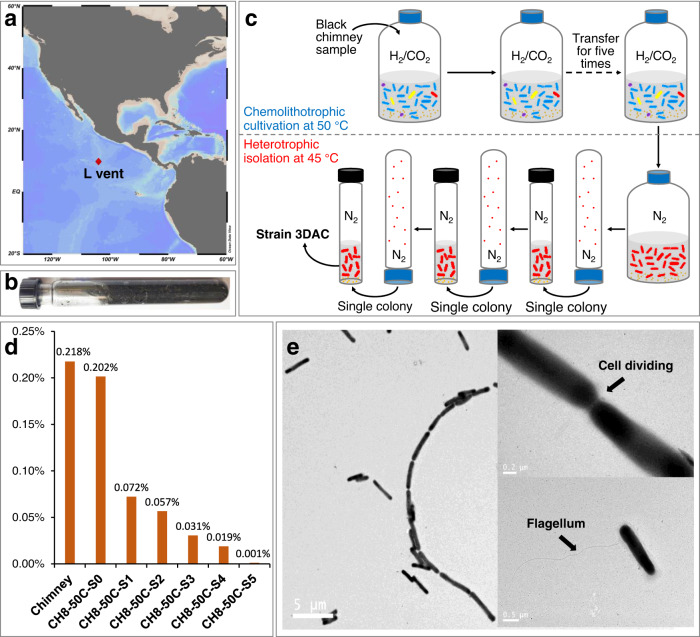
Strain isolation procedure. **a** Location of L-vent at the 9°N vent field on the East Pacific Rise. The map was generated by Ocean Data View (Schlitzer, Reiner, Ocean Data View, https://odv.awi.de, 2021). **b** The black L-vent chimney sample collected in an anaerobic tube. **c** Schematic diagram of the “Subtraction-Suboptimal” (StS) strategy used to isolate *Zhurongbacter thermophilus* strain 3DAC in this study. **d** The track of proportion changes of 3DAC closely related sequences in the chimney sample, original enrichment, and different transfers. Labels: Chimney, the chimney sample; CH8-50C-S0, original enrichment; CH8-50C-S1 to -S5, the first to fifth subcultures transferred from the original enrichment. All samples indicate that strain 3DAC represents a rare species. **e** Transmission electron micrograph of 3DAC, in which 3DAC cells form long filaments with chains of up to 11 cells or more; the micrograph showing a connection between two 3DAC cells; and a long lateral flagellum of 3DAC. The TEM experiment was repeated four times, and long filamentous patterns were observed each time. Source data are provided as a Source Data file.

To increase the probability to cultivate novel thermophilic bacteria from the rare biosphere, we designed a two-stage cultivation strategy, named “Subtraction-Suboptimal” (StS) strategy (Fig. 1c). The concept of this method is to reduce the complexity of the community and minimize the abundance of dominant heterotrophic organisms by using first an autotrophic culture medium and subculturing for several times (denoted “Subtraction”), followed by switching to a rich heterotrophic medium at a suboptimal temperature to recover the target heterotrophic thermophilic bacteria present in low abundance and to avoid competition from usually dominant fast-growing hyperthermophilic heterotrophic archaea (denoted “Suboptimal”).

In this case, the original sample, collected from an active black smoker chimney emitting hydrothermal fluid with a temperature of 231 °C (Fig. 1a, b; ref. ^34^), was firstly cultivated in a chemolithoautotrophic medium (SME medium, see “Methods”) at 50 °C, then consecutively transferred for five times under autotrophic condition, reducing the microbial community complexity and the ratio of heterotrophs. The conditions of the chemoautotrophic “Subtraction” stage mimicked the natural environment in situ and established a community dominated by autotrophs (mainly *Hydrogenimonas* within Campylobacterota and *Desulfurobacterium* within Aquificota)^35^. It performed similarly to the traditional enrichment method by decreasing the proportion of heterotrophs, but kept the co-cultured heterotrophs with autotrophs. In general, while the community is simplified through the “Subtraction” stage, it still maintains a certain degree of diversity, providing more opportunities for the isolation of rare species supported by autotrophs.

For the “Suboptimal” stage, we switched the cultivation condition from the autotrophic medium to a heterotrophic medium to provide a strong selection for heterotrophs. However, we decreased the cultivation temperature from 50 to 45 °C. While this resulted in a suboptimal growth temperature for thermophilic bacteria, it prevented the growth of heterotrophic hyperthermophilic archaea (i.e., *Thermococcus*, growth temperature ranges between 50 and 100 °C^36^), which were pervasive in the chimney sample, allowing the recovery of rare heterotrophic thermophilic bacteria. After a four-day incubation, growth was observed, followed by the isolation of single transparent and round colonies by using the rolling tube method^37^ containing a solid TRM medium.

During the “Subtraction” stage, some low-abundance heterotrophic species co-cultured with the autotrophs can be retained, these rare species can be hardly isolated by traditional methods when fast-growing heterotrophic microorganisms present at the same time. The successful isolation of the strain 3DAC, which represented only ~0.001% of the total community in the fifth transfer of the chemoautotrophic enrichment (~0.2% in the chimney sample, Fig. 1d), highlights the potential of the “StS” strategy to isolate rare species from environmental samples.

Here we provide a new idea of a two-step strategy to achieve the isolation of rare or unknown species that have a similar ecological niche to a dominant group. The “Subtraction” process makes a dilution-to-extinction within a community to realize the simplification of microbial composition, and the “Suboptimal” process recovers the rare species by the suboptimal condition that is more harmful to the dominant group in a similar ecological niche. This “StS” strategy may provide new opportunities to discover rare taxa hidden behind the common dominant taxa in studied environments.

#### Morphology and physiology

The cells of strain 3DAC are rod-shaped, 1.3–7.5 μm long (average 3.1 ± 1.3, *n* = 25) and 0.4–0.6 μm wide (average 0.5 ± 0.07, *n* = 25). They have a single lateral flagellum and are highly motile under a light microscope. During growth, the strain 3DAC occurs mostly as single cells, but sometimes forms long chains (Fig. 1e and Supplementary Fig. 1). The strain 3DAC grows only under strictly anaerobic conditions and performs as a piezophilic thermophile with: temperature range of 30–75 °C (optimal 70 °C), NaCl range of 1.0–4.5% (w/v) (optimal 2.5 %), pH range of 5.5–8.5 (optimal pH 7.0), and hydrostatic pressure range of 0.1–80 MPa (optimal 20 MPa) (Supplementary Fig. 2a–d).

#### Carbon source utilization

**Fig. 2:**
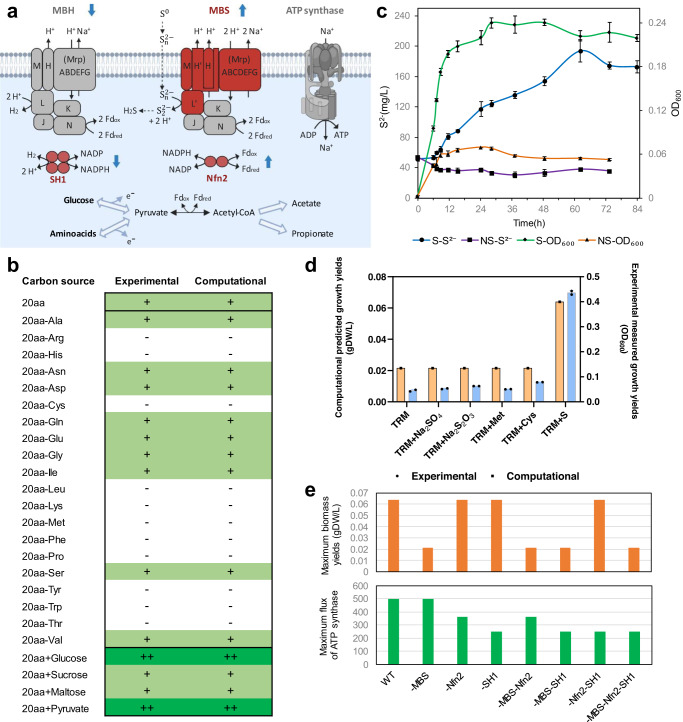
Energy metabolism of strain 3DAC. **a** Schematic diagram of the energetic process in strain 3DAC. Red shapes represent archaeal orthologs. Gray shapes represent bacterial orthologs. Solid lines represent enzymatic reactions. Dotted lines represent abiotic reactions. Differentially expressed genes were identified using DESeq2 (version 1.12.4). The default DESeq function with betaPrior = FALSE was applied, wherein a negative binomial generalized model with Wald test for significance was performed. The two-sided p-values were corrected for multiple testing using the Benjamini–Hochberg method. Blue arrows indicate significant up- or down-regulation with sulfur in the culture medium based on transcriptomic data [*n* = 3 per treatment, log2(fold change) >1, *P* value < 0.05] (Supplementary Data 1). This figure was created using BioRender (https://biorender.com/). **b** Growth capabilities on varied carbon sources of strain 3DAC. Computational predictions were simulated by the genome-scale metabolic model of 3DAC (Supplementary Data 1). “20aa”: all 20 amino acids supplemented as the sole carbon source. “20aa-X”: removal of individual amino acids X. “20aa+Y”: extra carbon source Y supplied with 20 amino acids. “+”: similar growth yields compared with the 20aa condition; “++”: at least 1.5-fold higher growth yields than 20aa; “-”: no growth. **c** Sulfide concentration and OD_600_ during the growth of strain 3DAC. S: medium with elemental sulfur; NS: no elemental sulfur. Error bars represent the standard deviations (SDs) from independent biological triplicates (*n* = 3) during the experiments under 0.1 MPa. Data are presented as average values ± SD. **d** Experimental and computational growth yields of the 3DAC strain by utilizing various sulfur species. In the experimental group, error bars represent the standard deviations (SDs) from independent biological duplicates (*n* = 2) during the experiments under 0.1 MPa. Data are presented as average values ± SD. **e** Simulations of wild-type 3DAC (WT), single deletions (-MBS, -Nfn2, -SH1), double deletions (-MBS-Nfn2, -MBS-SH1 and -Nfn2-SH1) and triple deletions (-MBS-Nfn2-SH1) of archaea-derived complexes in energetic processes at 3DAC. Source data are provided as a Source Data file.

Separately adding peptone, casamino acids, beef extract, yeast extract, and tryptone at a final concentration of 0.2% (w/v) supported the rapid growth of strain 3DAC (Supplementary Fig. 2e). In a basal TRM medium without a carbon source, no growth was detected upon separately adding only one type of any carbohydrate or organic acid (see “Methods”) under the tested conditions. However, strain 3DAC grew well with 20 canonical amino acids simultaneously added to the basal TRM medium. Moreover, “leave-one-out” experiments with 20 amino acids revealed that 3DAC relied on 11 essential amino acids (i.e., l-arginine, l-cysteine, l-histidine, l-leucine, l-lysine, l-methionine, l-phenylalanine, l-proline, l-threonine, l-tryptophan, and l-tyrosine) (Fig. 2b), no growth was observed as the absence of any one of these amino acids. In the presence of 20 amino acids, d-(+)-glucose and pyruvate can be used as additional carbon sources (Fig. 2b and Supplementary Data 1).

### Genomic and phylogenetic analyses indicate 3DAC as a deep-branching, sulfur-reducing heterotrophic bacterium

#### Genome characteristics

The complete genome of strain 3DAC is a single circular chromosome of 1,588,679 bp with a GC content of 41.15% (Supplementary Fig. 3). The 3DAC genome contains three rRNA gene operons and 46 tRNA genes. A total of 1524 coding DNA sequences (CDSs) with an average length of 980.78 bp, covering 94.09% of the whole genome, can be identified. The strain 3DAC has a complete glycolysis pathway, suggesting a capability of carbohydrate utilization, but many genes related to amino acid synthesis are absent, indicating the requirement of certain amino acids. This phenomenon is confirmed by the results of physiological experiments, the growth of 3DAC depends on the addition of amino acids and can be stimulated by adding carbohydrates (Fig. 2b). The loss of genes related to amino acid synthesis is common among thermophiles. Since the synthesis of amino acids consumes much energy^38^, it is more efficient for thermophiles to absorb amino acids directly from the environment, which is more conducive to their adaptation to the hydrothermal environment.

#### Archaea-derived energetic process in 3DAC

The respiratory energetic process in strain 3DAC contains a Na^+^-driven ATP synthase (Supplementary Fig. 4), a 12-gene cluster coding a membrane-bound hydrogen-producing complex (MBH) and a 13-gene cluster coding the membrane-bound sulfur respiration complex (MBS), which is similar to the energetic process in hyperthermophilic archaea within the order Thermococcales^39–41^ (Fig. 2a). MBS enables cells to reduce elemental sulfur (S^0^) to hydrogen sulfide (H_2_S) in vivo and to use reduced ferredoxin as an electron donor^40^. Genes in MBH of 3DAC, including the key catalytic subunit of hydrogen production (MbhL), are bacterial orthologs, as expected, while most of the MBS-coding genes (10 of 13) of 3DAC are archaeal orthologs encoding the complete membrane arm (MbsA, B, C, D, E, G, H, H’ and M subunits) and the key catalytic subunit of sulfur reduction (MbsL) (Fig. 2a and Supplementary Data 1). Compared to the bacterial hydrogen-producing MBH, the archaea-derived MBS enables cells to convert energy more efficiently with a 3.4-fold higher redox potential to reduce elemental sulfur (S^0^) to hydrogen sulfide (H_2_S) than MBH^39,40^. In addition, two redox-balancing complexes in 3DAC, soluble hydrogenase I (SH1) and NADP-dependent ferredoxin oxidoreductase II (Nfn2), are also archaeal orthologs with a high identity (>60%) as genes reported in hyperthermophilic archaea Thermococcales^42,43^.

Consistent with genomic analysis, physiological experiments revealed that the presence of elemental sulfur significantly stimulated the growth of strain 3DAC, compared with the medium without sulfur (Fig. 2c, d). Other sulfur substitutes, such as sodium sulfate, sodium thiosulfate, l-cysteine or l-methionine, did not significantly stimulate growth (Fig. 2d and Supplementary Data 1). During the growth of 3DAC, elemental sulfur is reduced to sulfide (Fig. 2c). This process is proposed to be conducted by MBS with the help of redox balancing via Nfn2 and/or SH1, which is also in agreement with transcriptomic analysis with or without elemental sulfur. We found that most genes (10 of 13) in MBS were significantly upregulated with elemental sulfur in the medium, including the catalytic subunit MbsL. Genes in Nfn2 were also highly expressed with sulfur, indicating the potential coupling of Nfn2 with MBS for redox balancing. In contrast, genes in MBH and SH1 were significantly downregulated in the presence of sulfur, which suggested competition between MBS and MBH and potential coupling between MBH and SH1 (Supplementary Data 1).

Furthermore, a genome-scale metabolic model of 3DAC, GEM-i3DAC (Supplementary Data 1), was constructed to simulate the impacts of the respiratory and redox-balancing complexes derived from archaea (i.e., MBS, SH1 and Nfn2) in 3DAC. The model incorporated both annotations from automatic pipelines and manually curated biochemical evidence of (hyper)thermophiles from the literature (Supplementary Data 1). This model was validated to predict growth trends accurately by utilizing varied carbon sources and electron acceptors compared to the experimental observations (Fig. 2b, d). Simulations were further performed on the 3DAC wild-type, single, double, and triple deletions of the archaea-derived complexes MBS, SH1 and Nfn2. Deletion of MBS blocked sulfur consumption and hydrogen sulfide production and theoretically decreased maximum biomass yields to 33% of the WT (Fig. 2e and Supplementary Data 1), which supported the necessity of MBS for utilizing sulfur as an electron acceptor to stimulate 3DAC growth. The single deletion of Nfn2 and SH1 decreased the maximum flux of ATP synthase to 72% and 50%, respectively, which indicated the essential function of Nfn2 and SH1 in ATP production. These results revealed the necessity of these archaea-derived complexes for both growth and ATP production in the 3DAC strain. Overall, strain 3DAC performed as a thermophilic heterotroph combining bacteria-derived hydrogen-producing and archaea-derived sulfur-reducing complexes for respiration. The ability of the thermophilic strain 3DAC to utilize the archaea-derived energy production might indicate its crucial evolutionary place.

#### Phylogenetic analyses identify 3DAC as a deep-branching lineage

**Fig. 3:**
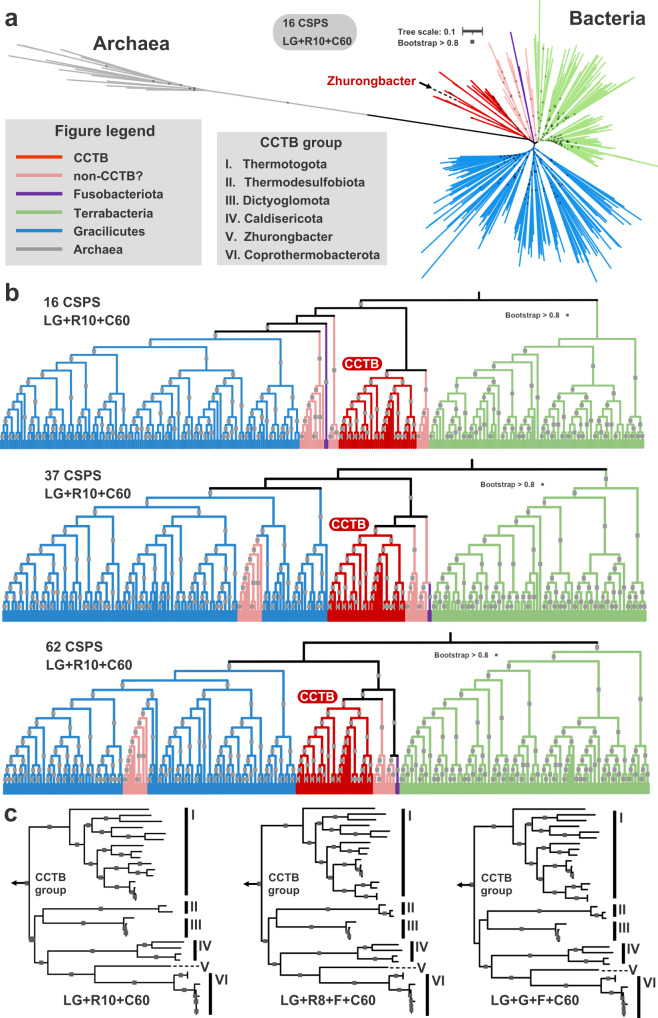
Phylogenetic analyses. **a** Phylogenomic tree of *Zhurongbacter thermophilus* 3DAC and other major thermophilic bacteria using Archaea as the outgroup with 16 conserved single-copy protein sequences (CSCP) with IQ-Tree using the LG + R10 + C60 model. **b** Phylogenomic tree of *Zhurongbacter thermophilus* 3DAC and other major thermophilic bacteria using 16/37/62 conserved protein sequences. Red color represents major thermophilic bacterial phyla, namely, Zhurongbacterota, Caldisericota, Coprothermobacterota, Dictyoglomota, Thermotogota, and Thermodesulfobiota. The pink color refers to Aquificota, Synergistota and Deinococcota, while the purple color refers to Fusobacteriota. **c** Phylogenomic inferences of the lineages from the CCTB with 37 conserved protein sequences with different models but without the Archaea genome. I-VI represent Thermotogota, Thermodesulfobiota, Dictyoglomota, Caldisericota, Zhurongbacterota, and Coprothermobacterota. The arrows indicate other bacterial branches. In all trees, the gray squares indicate a bootstrap higher than 0.8.

Phylogenomic analysis based on different concatenated conserved protein sequences^24,44–46^ and 16S rRNA gene-based analysis both place the phylogenetic position of strain 3DAC as a sister lineage to Coprothermobacterota, a bacterial phylum proposed by Pavan et al. (2018) ^47^ (Fig. 3a–c and Supplementary Fig. 5a–c). The most closely related cultured strain of 3DAC based on both 16S rRNA gene phylogenetic and phylogenomic analyses was *Coprothermobacter proteolyticus* DSM5265 but sharing a low 16S rRNA gene identities (81.25–83.00%) and AAI (48–49%) (Supplementary Data 3 and 4). *C. proteolyticus* DSM5265 is an anaerobic thermophilic microbe frequently identified in artificial thermophilic anaerobic systems, and grows better without sodium chloride and is able to reduce thiosulfate to sulfide^31,48–50^, while 3DAC was isolated from the natural hydrothermal vent and has a preference to utilize amino acid and elemental sulfur. Phylogenetic analysis of the 16S rRNA genes shows that strain 3DAC, together with other related 16S rRNA genes from enrichment cultures from tubes of *Alvinella pompejana* collected from a deep-sea vent at 13°N on the East Pacific Rise, forms a monophyletic cluster, clearly separating them from Coprothermobacterota (Supplementary Fig. 5c). We also compiled almost complete 16S rRNA gene sequences (longer than 1400 bp, >95% coverage) from Coprothermobacterota and compared the sequence identities with those of the 3DAC clade. The 16S rRNA gene sequence identities ranged from 75.73 to 83.00% (Supplementary Data 5). Since strain 3DAC was isolated from a high-temperature hydrothermal vent environment, we propose 3DAC as *Zhurongbacter thermophilus* 3DAC, named after Zhurong, the fire god in Chinese myth. For higher taxonomic classification, based on the suggested AAI ranges for the whole-genome^51^ and median 16S rRNA gene sequence^52^ identities to distinguish a new phylum, it is likely that this lineage represents a previously unknown thermophilic bacterial phylum. However, the relative evolutionary divergence (RED) score of strain 3DAC indicates that 3DAC might belong to a class-level lineage within the phylum Coprothermobacterota based on GTDB taxonomy^53^. There is a discordance between traditional classification and GTDB, hence, here we only temporally call the clade represented by 3DAC as a deep-branching lineage Zhurongbacterota until more 3DAC-related species are discovered for more accurate taxonomic assessment, as well as the prokaryotic taxonomic definition of phylum reach a consensus within the scientific community.

### Evolutionary history of the 3DAC-related thermophilic clade

#### Deep-branching thermophilic bacterial phyla cluster closely with 3DAC on phylogenomic trees

Phylogenetic analysis based on different sets of the concatenated conserved protein sequences with different inference models revealed that, in addition to Coprothermobacterota^31,47,50^, strain 3DAC is most closely related to other thermophilic bacterial groups, i.e., Caldisericota^54,55^, Dictyoglomota^56^, Thermotogota^16,57,58^, and Thermodesulfobiota^44,47,59–63^ (Fig. 3a–c). These results are consistent with previous phylogenetic studies employing different protein makers^55,64^, all found that these thermophilic bacterial lineages clustered together. Nevertheless, we noticed that the 16S rRNA gene-based phylogenetic tree places several non-thermophilic bacterial phyla within a clade, which we term “closely clustered thermophilic bacteria” (CCTB) (Supplementary Fig. 5a, b). The phylogenetic conflict between 16S rRNA gene and concatenated conserved protein sequences may be caused by the potential phylogenetic artifacts arising from a compositional bias of 16S rRNA gene, as have shown in previous studies^65–68^, which may not accurately reflect the evolutionary history of the thermophilic lineages. On the contrary, concatenated alignments of multiple universally distributed single-copy marker genes have become increasingly important in evolutionary analyses as they provide greater resolution and span a larger portion of the genome than individual marker sequences^69^.

We also tested their phylogenetic positions with two outgroup rooting datasets, i.e., using Archaea as an outgroup, and using bacterial root position proposed in ref. ^24^. The results indicate that these thermophilic groups consistently cluster near the root of the Archaea–Bacteria phylogenomic tree, or display deep-branching monophyly with the sister clade Terrabacteria by using the two rooting methods, respectively (Fig. 3a, b). Physiologically, all the above-mentioned bacterial lineages also share similar metabolic capabilities, for example, nearly all their lineages have an anaerobic lifestyle, are able to use elemental sulfur or sulfur-related compounds and are heterotrophs with the ability to degrade sugars or/and proteins (Supplementary Data 6). Therefore, here we refer to this clade as “closely clustered thermophilic bacteria (CCTB)” to describe these thermophilic bacterial phyla that cluster closely with 3DAC and branch together on bacterial phylogenomic trees. The CCTB contains at least six lineages that have pure cultured strains: *Zhurongbacter*, Caldisericota, Coprothermobacterota, Dictyoglomota, Thermotogota, and Thermodesulfobiota. Some other candidate phyla such as Deinococcota and Synergistota also cluster with the clade (Fig. 3a) and therefore might belong to the CCTB. However, although a number of their members are also thermophilic bacteria, these phyla have evolved multiple non-thermophilic bacteria lineages^70–72^. Besides, some candidate phyla cluster with the CCTB on the 16S rRNA gene tree but display some inconsistency on the phylogenomic tree using different conserved marker protein sequence sets, such as thermophilic Aquificota. We have carefully checked the phylogeny of Aquificota by different methods, and found that most of the genes within Aquificota have undergone horizontal gene transfer (HGT) as previously described^73,74^ (Supplementary Figs. 6 and 7), which would influence its phylogenetic inference. In general, further study regarding whether the phyla Deinococcota, Synergistota, Aquificota, and even other closely related clades had a thermophilic origin and branch as sister lineages with CCTB need to be resolved in the future.

#### Evolution and metabolic potentials of the predicted CCTB common ancestor

Phylogenetic ancestral analyses indicate that the members of the CCTB (Zhurongbacter, Caldisericota, Coprothermobacterota, Dictyoglomota, Thermotogota, and Thermodesulfobiota) might have shared one potentially last common ancestor. The predicted ancestral gene set from six lineages, in general, includes genes coding for thermophilic-related components (putative heat-shock proteins), flagellar that enables its movement, hydrogenase (FeFe group type) as well as saccharide and amino acid utilization pathways (ALE predicted gene copy number >0.3, Supplementary Data 2). Nevertheless, the amino acid biosynthesis pathways are not complete, possibly due to the limitation of ancestral prediction. A genome-scale metabolic model of the CCTB ancestor was further constructed and suggests it is a motile mixotrophic bacterium with carbon dioxide, saccharides and proteins as substrates (Supplementary Data 2). This predicted ancestral metabolism of the CCTB ancestor also shares many similarities with the recently inferred metabolic capacities of the last universal common ancestor (LUCA) and LBCA^20,24,75^. The CCTB ancestor, LUCA and LBCA are considered strictly anaerobic microorganisms. Both the CCTB ancestor and LUCA are predicted to be thermophilic and share the potential for metabolizing hydrogen, while LBCA and the CCTB ancestor share the predictions for motility and the potential for degrading organic carbon compounds.

**Fig. 4:**
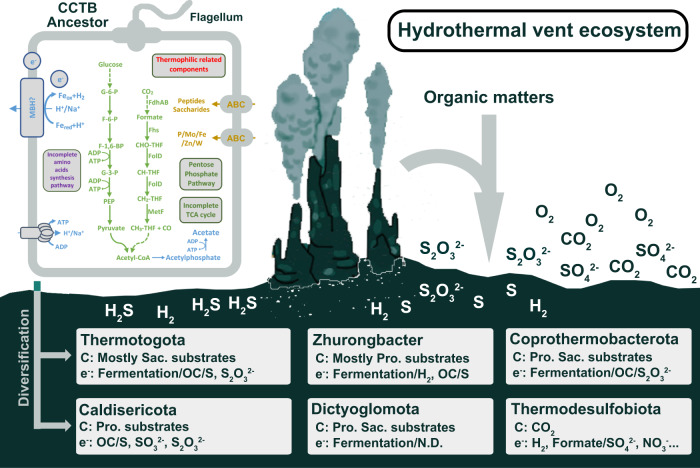
Metabolic capability of the CCTB ancestor and its diversification. Illustration of metabolic features of the CCTB ancestor and different bacterial lineages from the CCTB (Supplementary Data 6), the hydrothermal vent here indicates that these bacteria might have originated from similar environments on early Earth but under adaptation radiation during their evolutionary history. Pro proteins, Sac. saccharides, OC organic carbon.

Within the CCTB clade, after the diversification of the CCTB ancestor, each lineage adapted to different environments and gradually evolved to form specific features by gene gain and loss (Fig. 4 and Supplementary Fig. 8), especially on sulfur metabolism and carbon utilization pathways. For sulfur respiration, most lineages of the CCTB contained the putative catalytic subunit MbsL-encoding gene of the MBS and/or MBH complex-encoding gene cluster (Supplementary Fig. 9)^40^, which is responsible for sulfur and hydrogen metabolisms. As the sulfur respiration capability strongly stimulates the growth of 3DAC in the present study and other strains in the previous studies^76^, it indicates that their ancestor might originate from a sulfur compound-rich hydrothermal vent or hot spring environment^77,78^. For carbon assimilation, members of the clades Zhurongbacter, Caldisericota, Coprothermobacterota, Dictyoglomota and Thermotogota are considered as thermophilic heterotrophic bacteria, but they diversified their preferences for carbon source (Supplementary Fig. 8), such as gain and loss of genes for peptide and sugar transport and motility genes (Fig. 4).

As cultivation technologies develop rapidly, more deep-branching thermophilic bacteria clustered with the CCTB will be isolated, and comprehensively characterized through our newly designed cultivation strategy. The discovery of the present novel deep-branching bacterium strain 3DAC with the ability to utilize archaea-derived energy production can be viewed as a piece of the thermophile evolutionary puzzle. With more deep-branching lineages to be discovered in the future, the physiological and evolutionary history of the CCTB will enable us to see through the basic metabolism and niche expansion of early life.

## Methods

### Sample collection

The original sample was collected with the remotely operated deep-sea research vehicle (ROV) *Jason II* during R/V *Atlantis* research cruise AT26-10 (Dive 761) from a black smoker chimney at l-vent located at the 9°N deep-sea vent field on the East Pacific Rise (9°46.25’ N, 104°16.74’ W, water depth 2528 m). The temperature of the emitted fluid was 231 °C. More information about the fluid geochemistry and microbial communities in the chimney walls can be found in ref. ^34^. Pieces of the chimney were stored immediately upon retrieval in sterile anaerobic tubes, and the headspace in the tubes was filled with pure nitrogen gas with slight overpressure to prevent the entry of oxygen. The samples were then stored at 4 °C until enrichment in the laboratory.

### “Subtraction-Suboptimal” strategy-guided isolation

The outer layer of the black chimney sample (0.25 g) was first enriched anaerobically in an SME medium^79^, which contained the following components (L^−1^): NaCl, 20.0 g; MgSO_4_·7 H_2_O, 3.5 g; MgCl_2_·6 H_2_O, 2.75 g; KCl, 0.325 g; KNO_3_, 2.0 g; MES, 4.27 g; NaBr, 50.0 mg; H_3_BO_3_, 15.0 mg; SrCl_2_·6 H_2_O, 7.5 mg; (NH_4_)_2_SO_4_, 10·0 mg; KI, 0.05 mg; Na_2_WO_2_·2H_2_O, 0.1 mg; CaCl_2_·2H_2_O, 0.75 g; KH_2_PO_4_, 0.5 g; NiCl_2_·6H_2_O, 2.0 mg; and resazurin, 1.0 mg. The pH of the medium was adjusted to 6.0 using 1 M NaOH. After autoclaving, 1 ml of trace element mixture, 1 ml of vitamin mixture, 1 ml of thiamine solution, and 1 ml of vitamin B12 solution^80^ were added to the medium (L^−1^). Then, 50 ml aliquots of the medium were separated into 150-ml glass serum bottles and supplemented with 0.5 g of elemental sulfur and 0.5 ml of Na_2_S·9H_2_O (10 %, w/v; pH 7.0), and the tightly stoppered bottles were pressurized with hydrogen/carbon dioxide (80: 20; 101 kPa). The cultures were incubated at 50 °C. We obtained the original enrichment in this chemolithotrophic medium, subcultured (1% inoculation) the cells in this medium for five generations, and then transferred the enrichment of the fifth generation (1% inoculation) to a heterotrophic TRM medium^81^ with the following composition (L^−1^): 1 g of yeast extract, 4 g of tryptone, 23 g of NaCl, 5 g of MgCl_2_·6H_2_O, 0.7 g of KCl, 0.5 g of (NH_4_)_2_SO_4_, 3.3 g of PIPES disodium salt, 1 ml of NaBr (5%, w/v), 1 ml of SrCl_2_·6H_2_O (1%, w/v), 1 ml of KH_2_PO_4_ (5%, w/v), 1 ml of K_2_HPO_4_ (5%, w/v), 1 ml of CaCl_2_·2 H_2_O (2%, w/v), and 1 ml of Na_2_WO_4_ (10 mM). The medium was adjusted to pH 7.0, autoclaved, separated into anaerobic tubes (5 ml), and supplemented with 0.05 g of elemental sulfur and 0.05 ml of Na_2_S·9 H_2_O (10 %, w/v; pH 7.0), and the headspace of the tubes was pressurized with pure nitrogen gas. Heterotrophic enrichment was performed at 45 °C. Colonies were obtained on solid TRM medium (1.5 % Gelrite) using the rolling tube method, which was repeated three times.

### Epifluorescence microscopy and transmission electron microscopy

Fluorescent staining by SYBR Green I (Invitrogen) was carried out on cells filtered onto 0.2-μm polycarbonate GTBP membranes (Millipore) following established protocols^82^. Images were captured with a fluorescence microscope (Nikon Eclipse 90i).

For transmission electron microscopy, cells were harvested at 3000 × *g* for 5 min, fixed in 4% formaldehyde for 2 h at room temperature, and washed with 1× PBS (pH 7.4). Fixed cells were negatively stained with 2% (w/v) phosphotungstic acid (pH 6.5) for observation with a transmission electron microscope (FEI Tecnai G2 Spirit Biotwin) at an acceleration voltage of 120 kV.

### Growth characteristics

The effects of the temperature, pH, and NaCl concentration on the growth of strain 3DAC were determined in an anaerobic TRM medium with a headspace gas composed of pure nitrogen gas at atmospheric pressure supplemented with 1% (w/v) elemental sulfur. To evaluate the optimum temperature for growth, strain 3DAC was cultivated at 25–80  °C with 5  °C intervals. The ranges of pH and NaCl concentrations for growth were determined at 45  °C. The optimal pH for growth was determined by varying the pH in the culture medium between 4.0 and 9.0 using the following buffers at a concentration of 10 mM: acetate at pH 4.0, 4.5, and 5.0; MES at pH 5.5 and 6.0; PIPES at pH 6.5 and 7.0; HEPES at pH 7.5 and 8.0; and TAPS at pH 8.5 and 9.0. The pH was adjusted at room temperature before autoclaving. To test the effect of the NaCl concentration on growth, NaCl was added to the medium at final concentrations within the range of 0.5–5.0% (w/v). All of these tests were performed in triplicate in the dark without shaking. Growth was determined by monitoring the OD_600_ with a quartz colorimeter (50 mm optical path) on a spectrophotometer (HACH DR5000). The specific growth rate (μ; h ^−1^) was calculated according to the equation μ = ln[OD_600(_*t*_2)_/OD_600(_*t*_1)_]/(*t*_2_ − *t*_1_), where OD_600(_*t*_1)_ and OD_600(_*t*_2)_ are the optical densities of liquid cultures measured at 600 nm at time points *t*_2_ and *t*_1_, and *t*_1_ and *t*_2_ are the time points (in hours) of the exponential growth phases^83^. Growth rates were calculated using linear regression analysis of 4 to 6 points along the linear portions of the growth curves.

### Determination of growth pressure

The pressure range (0.1, 10, 20, 30, 40, 50, 60, 70, 80, 90, and 100 MPa) was tested with syringes loaded with TRM (2.5% NaCl, pH 7.0) supplemented with 1% (w/v) elemental sulfur. These syringes were placed into high-pressure and high-temperature reactors and incubated at 70 °C. Growth yields and growth rates were measured as previously described. All of these tests were performed in triplicate.

### Essential amino acid tests

Mother liquors of 20 kinds of amino acids (l-alanine, l-arginine, l-asparagine, l-aspartic acid, l-cysteine, l-glutamine, l-glutamic acid, glycine, l-histidine, l-isoleucine, l-leucine, l-lysine, l-methionine, l-phenylalanine, l-proline, l-serine, l-threonine, l-tryptophan, l-tyrosine, and l-valine) were prepared. Then, each combination of 19 amino acid mother liquors was added to basal TRM medium (2.5% NaCl, no yeast extract or tryptone, supplemented with 1 ml of trace element solution^84^, 10 ml of vitamin solution^85^ and 1% (w/v) elemental sulfur) to maintain each amino acid at a final concentration of 0.1 g/L. Basal TRM medium containing all 20 amino acids and no amino acids was set as the positive and negative controls, respectively. The pH of all the tested media was adjusted to 7.0 at room temperature. For all tests, positive cultures were transferred (1% inoculum) into the tested medium at least five times for confirmation of growth. All tests were performed in duplicate at atmospheric pressure and with incubation at 70 °C.

### Determination of growth requirements

Various single carbon sources were added to a basal TRM medium (2.5% NaCl, containing only 0.002% yeast extract, without any other organic carbon sources) supplemented with 1% (w/v) elemental sulfur. The following single carbon sources, all at a final concentration of 0.2% (w/v), were tested: peptone, casamino acids, beef extract, yeast extract, tryptone, sodium acetate, sodium formate, sodium citrate, sodium pyruvate, sodium oxalate, d-fructose, d-(+)-glucose, d-(+)-xylose, d-(+)-maltose, sucrose, starch, glycogen, N-acetyl-d-glucosamine, d-mannitol, l-alanine, l-arginine, l-asparagine, l-aspartic acid, l-cysteine, l-glutamine, l-glutamic acid, glycine, l-histidine, l-isoleucine, l-leucine, l-lysine, l-methionine, l-phenylalanine, l-proline, l-serine, l-threonine, l-tryptophan, l-tyrosine, and l-valine. Growth was recorded after 24 and 48 h. Negative (uninoculated basal TRM) and positive (inoculated TRM containing yeast extract and peptone) controls were prepared for each substrate. For all tests, positive cultures were transferred (1% inoculum) into the tested medium for confirmation of growth.

To test carbon source utilization with the addition of 20 kinds of amino acids, various carbon sources at a final concentration of 0.2% (w/v) were added into a basal TRM medium (2.5% NaCl, no yeast extract or tryptone) and supplemented with 20 kinds of amino acids (each at a final concentration of 0.1 g/L) and 1% (w/v) elemental sulfur. The carbon sources used were as follows: d-(+)-glucose, d-fructose, d-(+)-maltose, sucrose, starch, sodium acetate, sodium formate, and sodium pyruvate. Basal TRM medium supplemented with 20 kinds of amino acids and 1% (w/v) elemental sulfur was used as a control.

To examine the ability of the isolate to grow in the absence of elemental sulfur, cells were cultured in TRM medium (2.5% NaCl, sodium sulfate removed) without sulfur. Other sulfur substitutes, such as sodium sulfate (20 mM), sodium sulfite (20 mM), sodium thiosulfate (20 mM), l-cysteine (20 mM) and l-methionine (20 mM), were also tested.

The pH of all the tested media was adjusted to 7.0 at room temperature. All tests were performed in duplicate at atmospheric pressure and with incubation at 70 °C. In cultures containing insoluble substrates, growth was monitored by microscopic examination.

The sulfide concentration was measured by the methylene blue method^86^ using a sulfide measurement kit (HACH) on a spectrophotometer (HACH DR5000).

### Genome sequencing and analysis

Genomic DNA was extracted by a modified SDS-based DNA extraction method^87^ and sequenced on the PacBio RSII SMRT and Illumina HiSeq X Ten sequencing platforms (BGI, China). The genome was assembled into one contig from the PacBio reads (3668 Mbp, 2308-fold coverage) by Falcon v0.3.0^88^ and Celera Assembler v8.3^89^. Then, the assembled genome was corrected with Illumina HiSeq data (736 Mbp, 463-fold coverage) using GATK v1.6–13^90^. Protein-coding genes were predicted by Glimmer v3.02^91^, and rRNA and tRNA annotations were performed with RNAmmer v1.2^92^ and tRNAscan-SE v1.3.1^93^, respectively. Predicted gene functions were annotated with databases including NCBI-nr (updated on Oct 10, 2017), KEGG (BlastKOALA, v2.2, updated on May 15, 2019)^94^, and eggNOG-Mapper v2.0.1b-2-g816e190^95^. Average amino acid identity (AAI) values were calculated by CompareM v.0.0.23 (https://github.com/dparks1134/CompareM).

### Amplicon sequencing and diversity analyses

For the chimney sample and chemoautotrophic enrichment, the bacterial V4 region of the 16S rRNA gene was amplified by B515F (5’-XXXXXXXXGTGCCAGCMGCCGCGGTAA-3’) with eight-nucleotide key tags for each sample and B806R (5’-GGACTACHVGGGTWTCTAAT-3’)^96^; the X region represents the various key tags for each sample. The PCR program was 3 min at 95 °C, followed by 35 cycles of 94 °C for 40 s, 56 °C for 1 min, and 72 °C for 1 min. The final extension step was 72 °C for 7 min. The 50 µL amplification mixture contained 1 µL of each forward and reverse primer, 1 µL template DNA, 5 µL 10× ExTaq buffer, 0.5 µL ExTaq polymerase (TaKaRa, Japan), 4 µL of 2.5 mM dNTP mix and 37.5 µL ddwater. PCR products were purified with an E.Z.N.A. Gel Extraction Kit (OmegaBio-Tek, USA) and sequenced on the MiSeq platform (Illumina, USA) according to the manufacturer’s instructions.

Data analysis of the 16S rRNA MiSeq sequences was performed using the QIIME version 1.9.1 software pipeline^97^ and the QIIME-compatible version of the SILVA-132 database for template-based alignment and taxonomic assignment. Assembled reads that passed the chimera check were clustered into de novo operational taxonomic units (OTUs) at a cutoff of 97% sequence similarity.

### RNA sequencing and quantitative real-time PCR

Cells of strain 3DAC cultured with and without sulfur were harvested at the log phase by centrifugation. Three replicates under each condition were prepared for transcriptome analysis. Total RNA was extracted using a Total RNA Extractor (TRIzol) kit (Sangon Biotech, China) according to the manufacturer’s protocol. For RNA sequencing library preparation, genomic DNA was removed with DNase I, and ribosomal RNA (rRNA) was removed with a Ribo-off rRNA Depletion Kit (bacteria) (Vazyme Biotech Co., Ltd, China). A complementary DNA (cDNA) library was generated by a VAHTS™ Stranded mRNA-seq Library Prep Kit (Vazyme Biotech Co., Ltd, China) following the manufacturer’s instructions. Sequencing was performed on the Illumina HiSeq 2500 (Illumina, USA) platform at Sangon Biotech (Shanghai Co., Ltd., China).

The sequencing quality controls of the raw reads produced by Illumina HiSeq 2500 were assessed using FastQC (https://www.bioinformatics.babraham.ac.uk/projects/fastqc/), and all samples passed. Low-quality reads (Q value of <20), ambiguous “N” nucleotides, adapter sequences and fragments of <35 bp were removed from raw reads with Trimmomatic^98^ for further analysis. rRNA was removed using a sortmerna^99^ with default settings. Read mapping of the non-rRNA to the genome of *Zhurongbacter thermophilus* 3DAC was performed using Bowtie 2^100^, and the relevant mapping information was summarized. The uniquely mapped reads were collected and analyzed with the DESeq2 package based on the Poisson distribution to identify the differentially expressed genes (DEGs). Estimation of gene expression levels was based on the transcript per million (TPM) values^101^.

To validate the RNA-Seq data, the expression levels of ten randomly selected genes were quantified using quantitative real-time PCR (qRT-PCR). After the extraction of RNA, residual DNA was removed by a TURBO DNA-free Kit (Thermo Fisher Scientific, USA), and cDNA synthesis reverse transcription was followed by a RevertAid First Strand cDNA Synthesis Kit (Thermo Fisher Scientific, USA). The primers used for quantitative real-time PCR (RT-qPCR) were designed by Primer-BLAST^102^ (http://www.ncbi.nlm.nih.gov/tools/primer-blast) and are listed in Supplementary Data 7. Gene expression was quantified in different samples using the 16S rRNA gene as a reference gene. RT-qPCR was performed on a StepOnePlus Real-Time PCR instrument (Applied Biosystems, USA) with PowerUp SYBR Green Master Mix (Applied Biosystems, USA) using the following program: 95 °C for 10 min, followed by 40 cycles of 95 °C for 15 s and 60 °C for 1 min. All qRT-PCR analyses were performed with four technical replicates. The RNA-Seq fold changes were plotted against the qRT-PCR fold changes, and the correlation coefficients (R^2^) between these two datasets were calculated (Supplementary Fig. 10).

### Phylogenetic analyses

For the 16S rRNA gene tree, the 16S gene sequences were aligned using SINA v1.2.11^103^, and the full alignment was stripped of columns containing 70% or more gaps with a Perl script. The maximum-likelihood phylogeny of the 16S rRNA gene tree was inferred by IQ-TREE^104^ v2.0.6 with the “-MFP -B 1000” options for best-fit model selection and ultrafast bootstrap. The reference sequences of other bacteria were downloaded from the Silva database, sequences smaller than 1400 bp were filtered out, and OTUs were picked from the remaining sequences at a cutoff of 97%. The above-described representative OTU sequences were used for the tree.

For phylogenomic analysis based on conserved proteins, the representative bacterial dataset used by Coleman et al.,^24^ and selected archaeal reference genomes from the different phyla were downloaded from the NCBI prokaryotic genome database (https://www.ncbi.nlm.nih.gov/assembly/). These reference genomes and the 3DAC genome from this study were used to construct a phylogenomic tree based on a concatenated alignment of three different sets of marker genes (Supplementary Data 8–10)^24,45,46^. Specifically, each of the marker protein sequences from the reference genomes and the 3DAC genome was aligned using the MAFFT algorithm v7.313^105^ with the parameters --ep 0 --genafpair --maxiterate 1000 and filtered with trimAl v1.4.rev2^106^ with the parameter -automated1. Then, all marker protein sequences were concatenated into a single alignment, and phylogenetic trees were built using IQ-Tree^104^ v2.0.6 with the best-fitting model LG + C60 + R10 with an ultrafast bootstrap value of 1000.

To illustrate the phylogenetic affiliation of the phylum Aquificota, reference genomes were downloaded from  the NCBI database, and 5-10 genomes were downloaded for each phylum according to the NCBI taxonomy. Then each of the 37 marker protein sequences from the reference genomes was extracted and aligned using the MAFFT algorithm v7.313 with the parameters --ep 0 --genafpair --maxiterate 1000 and filtered with trimAl v1.4.rev2 with the parameter -automated1. Then 37 marker protein sequences were separately used to build phylogenetic trees using IQ-Tree v2.0.6 with the model LG + C60 + F + G with an ultrafast bootstrap value of 1000 to evaluate the potential HGT events.

For the phylogenetic analysis of the MbsL protein, its sequences were extracted from the reference genomes of Caldisericota, Thermotogota, Thermodesulfobiota, Aquificota, Zhrongbacter, and Thermococcales. These sequences with references were then aligned using the MAFFT algorithm v7.313 with the parameters --ep 0 --genafpair --maxiterate 1000 and filtered with trimAl v1.4.rev2 with the parameter -automated1. After that, the phylogenetic tree was built using IQ-Tree v2.0.6 by the best-fitting model with an ultrafast bootstrap value of 1000. All trees were visualized by iTOL^107^ online software.

### Evolutionary analyses of the CCTB

A total of 43 cultured bacterial complete genomes (Supplementary Data 11) from the CCTB, as well as potential candidate phyla were selected for evolutionary analyses. Genomes were downloaded from NCBI genomic database and predicted with Prodigal version 2.6.3^108^. Then all genomes were annotated with eggNOG-Mapper v2.0.1b-2-g816e190^95^. The comparative genomic analysis was performed by OrthoFinder^109^ with default parameters. A total of 6,578 orthogroups from 43 representative genomes were obtained from OrthoFinder^109^. The orthogroups that contain four or more sequences were separately aligned using the MAFFT algorithm v7.313^105^ with the parameters --ep 0 --genafpair --maxiterate 1000, filtered with trimAl v1.4.rev2^106^ with the parameter -automated1 and then trees were constructed using IQ-Tree with the best-fitting model. To address the evolutionary history of the CCTB, ancestral family gene sets were inferred using the program amalgamated likelihood estimation (ALE) version 0.4^110^. Only bacteria with complete genome information were considered in the present study because incomplete genomes would lead to biased analyses in gene gain and loss processes. Here, the phyla Synergistota and Deinococcota were used as the outgroups. The representative genomes from the phyla Aquificota, Thermosulfidibacterota and Thermodesulfobacteriota were also considered in the ALE analysis. For the CCTB, cultured bacteria with complete genomes in the phylum Dictyoglomota includes *Dictyoglomus turgidum* DSM6724 and *Dictyoglomus thermophilum* H-6-12; the phylum Thermodesulfobiota includes Thermodesulfobium acidiphilum 3127-1 GCA and Thermodesulfobium narugense DSM14796; the phylum Caldisericota includes *Caldisericum exile* AZM16c01; the phylum Zhurongbacter includes *Zhurongbacter thermophilus* 3DAC; the phylum Coprothermobacterota includes *Coprothermobacter platensis* DSM11748 and *Coprothermobacter proteolyticus* DSM5265; and the phylum Thermotogota includes *Pseudothermotoga hypogea* DSM11164, *Thermotoga neapolitana* DSM4359, *Thermosipho melanesiensis* BI429, *Fervidobacterium pennivorans* DSM9078, *Kosmotoga pacifica* SLHLJ1, *Mesotoga prima* MesG1Ag42, *Defluviitoga tunisiensis* L3, *Petrotoga mobilis* SJ95, *Marinitoga piezophila* KA3, and *Athalassotoga saccharophila* NAS-01. Ancestor metabolic reconstruction was performed with the predicted ancestral gene set (851 sequences) at the node of the CCTB with an average gene copy number larger than 0.3.

### Metabolic modeling

A genome-scale metabolic model of *Zhurongbacter thermophilus* 3DAC, GEM-i3DAC, was constructed according to the complete genome of the 3DAC strain. The draft model construction referred to ortholog mapping to public databases (KEGG^111^, EggNOG^95^, BIGG^112,113^, ModelSeed^114^, and TCDB^115^) (performed in November 2020) and the published model of the related thermophilic bacterial strain *Thermotoga maritima* MSB8^116,117^. Extensive manual curations were carried out following draft model reconstruction to integrate the latest biochemical evidence of enzymatic functions in (hyper)thermophiles. Overall, literature evidence was assigned to 57 reactions in the 3DAC model. The biomass equations of 3DAC were formulated based on the biosynthesis of macromolecules, including DNA, RNA, and proteins. Their biosynthesis was defined to account for the millimole composition of each building block in assembling one gram of a given component and the associated energy cost. Simulations of carbon source utilization, electron acceptors, and gene deletion were formulated with exchange constraints that represent the corresponding culture conditions used in the experiments.

The KO terms of the predicted ancestral gene set were used to construct the initial genome-scale metabolic model of the last common ancestor of the CCTB. Reactions for each KO term were obtained from the latest KEGG database (performed in March 2022). Compounds were initially introduced from the latest KEGG database, but the physiological charges of each and the modified hydrogen number of charged compounds were added. The biomass objective function was introduced from the *Escherichia coli* core model^118^, including DNA, RNA, and proteins that accounted for ~80% of the dry weight of cells^119^ (Supplementary Data 2). Metabolic simulations were performed with exchange constraints identified based on the environmental parameters measured in typical hydrothermal vents^120^ and the potential nutrients and products revealed by metabolic reconstruction (Supplementary Data 2). The current knowledge of amino acid synthesis pathways in bacteria and archaea based on gene annotation is inadequate, and amino acids that are missing or incomplete in the synthesis pathway may also be nonessential^121,122^. 3DAC have very similar metabolic characteristics to *Thermococcus*, and based on our previous results of essential amino acid testing for *Thermococcus eurythermalis* A501 and the presence/absence of their key enzymes in the complete genome^123^, we have delineated a criterion for determining essential amino acids at the genomic level, trying to reduce the bias caused by this knowledge gap. Essential amino acids were identified as those missing 50% or more of the reactions from the whole biosynthesis pathway. Other amino acids were treated as nonessential. To quantitatively simulate the growth yields using the tested sole carbon sources, each carbon source was constrained to 10 mM of total carbon to avoid the influence of different carbon numbers in the formula, with all essential amino acids (1 mM for each).

For both the 3DAC model and the ancestor model, consistency checks of all reactions were computationally conducted using the *formulacheck, chargecheck* and *masscheck* functions in PSAMM version 1.1.1 (https://zhanglab.github.io/psamm/)^124^, and the unbalanced equations were manually curated. Gap-filling processes were performed to complete the synthesis of the main biomass components (DNA, RNA, and proteins) and essential cofactors (i.e., NAD^+^/NADP^+^ and CoA) by the *fastgapfill* function in PSAMM. Flux variation analysis (FVA) to optimize biomass production was performed using the *fva* function in PSAMM with IBM ILOG CPLEX Optimizer version 12.7.1.0.

### Reporting summary

Further information on research design is available in the Nature Portfolio Reporting Summary linked to this article.

## Supplementary information

Supplementary

Description of Additional Supplementary Files

Supplementary Data 1

Supplementary Data 2

Supplementary Data 3-11

Reporting Summary

Source_data

## Data Availability

The genome sequence of *Zhurongbacter thermophilus* 3DAC was reconstructed and deposited under accession number CP046447. Amplicon sequencing data have been deposited at SRA under accession numbers SRR14072822, SRR14072823, SRR14072817, SRR14072825, SRR14072824, SRR14072798, and SRR14072797, and transcriptome sequence data have been deposited at SRA under accession numbers SRR14072890 and SRR14072891. Source data are provided with this paper.
