# Supplementary material for: Identification of a deep-branching thermophilic clade sheds light on early bacterial evolution
Source: Nat Commun. 2023 Jul 19;14:4354. doi: 10.1038/s41467-023-39960-x (PMC10356935; doi:10.1038/s41467-023-39960-x)
Supplement: Supplementary file 1 — Supplementary [file 41467_2023_39960_MOESM1_ESM.pdf]

Supplementary Information for

Identification of a deep-branching thermophilic clade sheds light on early bacterial evolution

Hao Leng[1,2,5], Yinzhao Wang[1,2,5], Weishu Zhao[1,2], Stefan M. Sievert[3], Xiang Xiao[1,2,4]*

[1]State Key Laboratory of Microbial Metabolism, School of Life Sciences and Biotechnology, Shanghai Jiao Tong University; Shanghai, China.

[2]International Center for Deep Life Investigation (IC-DLI), Shanghai Jiao Tong University; Shanghai, China.

[3]Biology Department, Woods Hole Oceanographic Institution; Woods Hole, MA, USA.

[4]Southern Marine Science and Engineering Guangdong Laboratory (Zhuhai); Zhuhai, Guangdong, China.

[5]These authors contributed equally to this work.

*Corresponding author. Email: zjxiao2018@sjtu.edu.cn

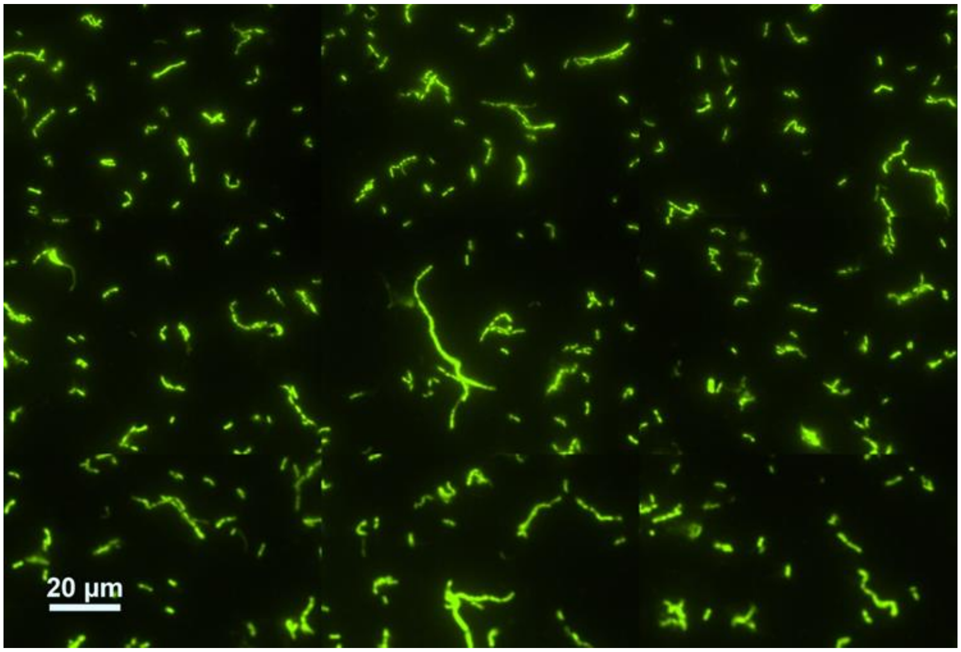

Supplementary Figure 1 | Fluorescence image of 3DAC cells stained with SYBR Green I. During growth, 3DAC cells formed long chains. The fluorescence imaging experiment was performed only once, because fluorescence imaging could not provide much detailed information, so more morphological observations were carried out under transmission electron microscope.

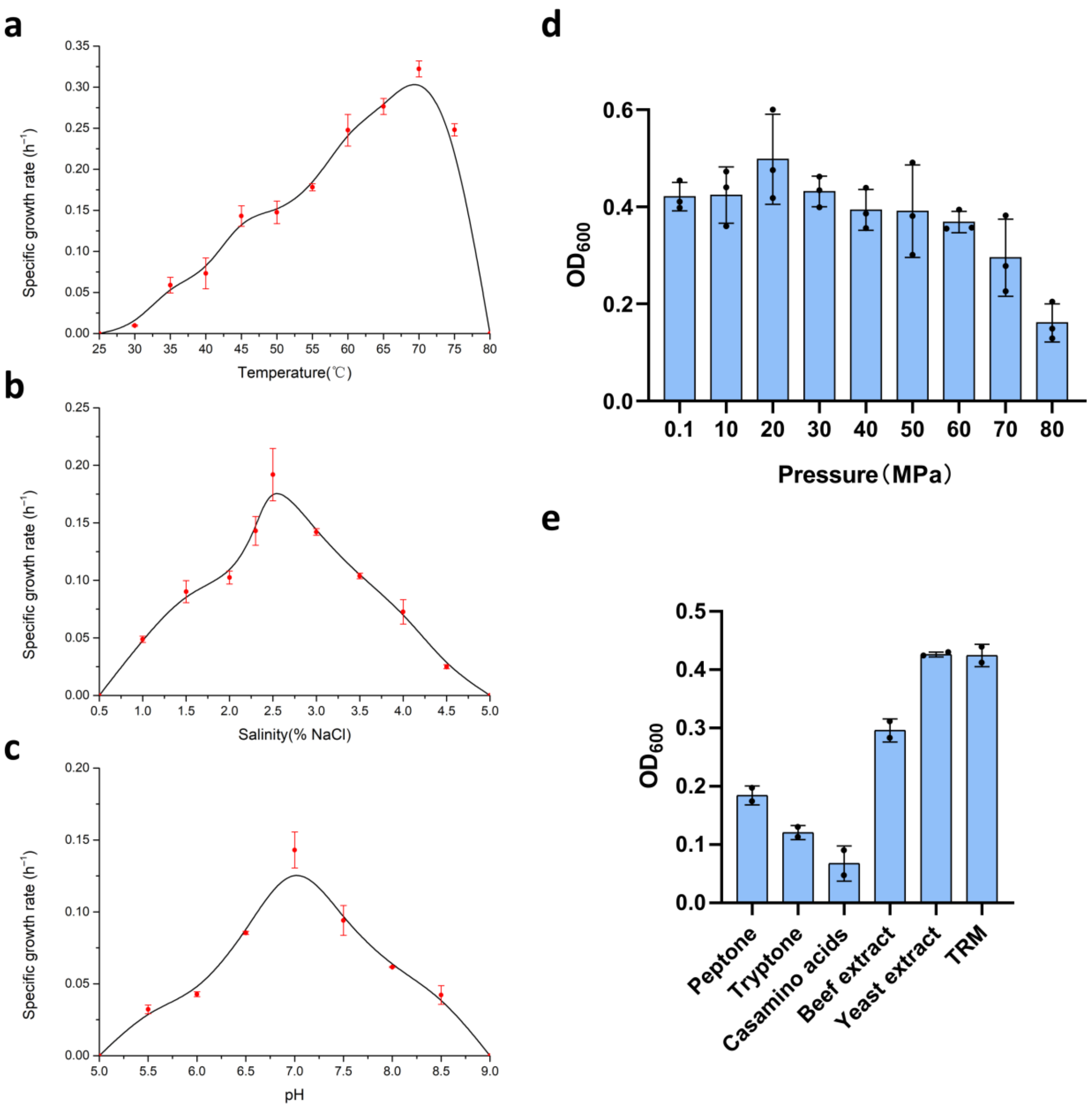

Supplementary Figure 2 | Overall characteristics of the 3DAC strain. Effects of the temperature (a), NaCl concentration (b), and pH (c) on the specific growth rate of strain 3DAC in TRM medium under atmospheric pressure. (d) Growth yields of strain 3DAC after 24 h incubations at different pressures. (e) Growth yields of strain 3DAC after 24 h incubations with different carbon sources. In Figure S2a-d, error bars represent the standard deviations (SDs) from independent biological triplicates (n = 3). In Figure S2e, error bars represent the standard

deviations (SDs) from independent biological duplicates (n = 2). All the data are presented as average values ± SD. Source data are provided as a Source Data file.

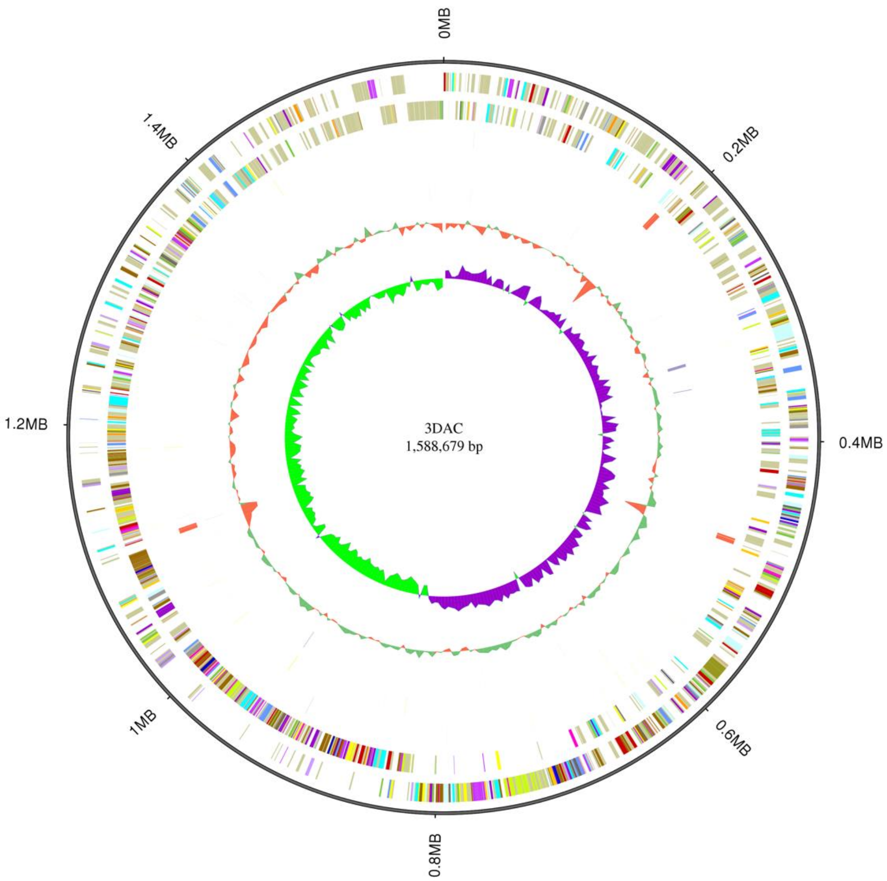

Supplementary Figure 3 | Circular representation of the strain 3DAC genome. Circles from the outside to the center: 1, genome size; 2, forward strand gene; 3, reverse strand gene; 4, forward strand ncRNA; 5, reverse strand ncRNA; 6, repeat; 7, GC content; 8, GC skew.

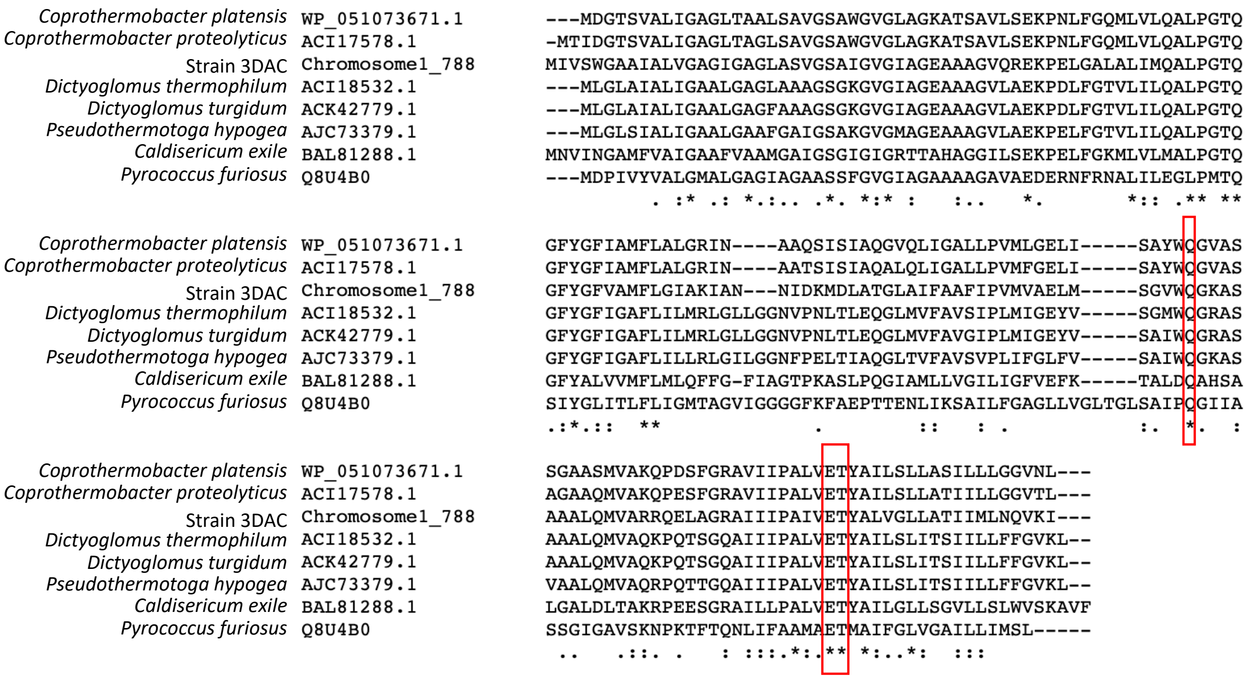

Supplementary Figure 4 | Multialignment of the c subunit in V-type ATP synthase of strain 3DAC and related species. The red boxes highlight the Na^+ - binding motif (“Q... ET” motif) in the c subunit. *Pyrococcus furiosus* was used as a reference. Sequence alignment was performed using Clustal Omega (Dublin, Ireland).

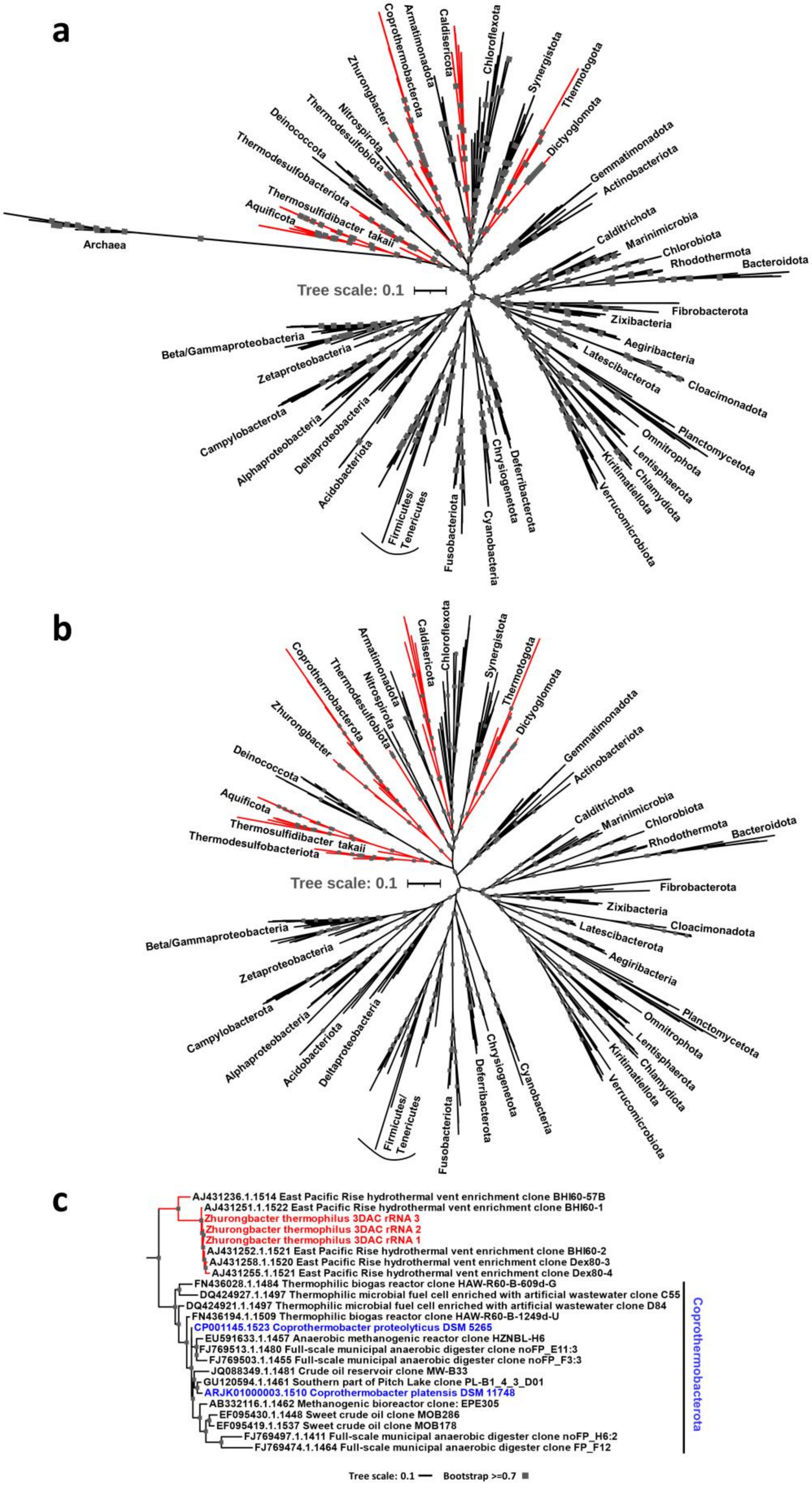

a
Coprothermobacterota
Armatimonadota
Caldisericota
Chloroflexota
Synergistota
Thermotogota
Dictyoglomota
Zhurongbacter
Nitrospirota
Thermodesulfobiota
Deinococcota
Thermodesulfobacteriota
Thermosulfidibacter takaii
Aquificota
Archaea
Gemmatimonadota
Actinobacteriota
Calditrichota
Marinimicrobia
Chlorobiota
Rhodothermota
Bacteroidota
Tree scale: 0.1
Fibrobacterota
Zixibacteria
Aegiribacteria
Latescibacterota
Cloacimonadota
Beta/Gammaproteobacteria
Zetaproteobacteria
Campylobacterota
Alphaproteobacteria
Deltaproteobacteria
Acidobacteriota
Firmicutes/Tenericutes
Fusobacteriota
Cyanobacteria
Chrysiogenetota
Deferribacterota
Verrucomicrobiota
Kiritimatiellota
Chlamydiota
Lentisphaerota
Omnitrophota
Planctomycetota
b
Coprothermobacterota
Armatimonadota
Caldisericota
Chloroflexota
Synergistota
Thermotogota
Dictyoglomota
Thermodesulfobiota
Nitrospirota
Zhurongbacter
Deinococcota
Aquificota
Thermosulfidibacter takaii
Thermodesulfobacteriota
Gemmatimonadota
Actinobacteriota
Calditrichota
Marinimicrobia
Chlorobiota
Rhodothermota
Bacteroidota
Tree scale: 0.1
Fibrobacterota
Zixibacteria
Cloacimonadota
Latescibacterota
Aegiribacteria
Planctomycetota
Omnitrophota
Lentisphaerota
Chlamydiota
Kiritimatiellota
Verrucomicrobiota
Beta/Gammaproteobacteria
Zetaproteobacteria
Campylobacterota
Alphaproteobacteria
Acidobacteriota
Deltaproteobacteria
Firmicutes/Tenericutes
Fusobacteriota
Deferribacterota
Chrysiogenetota
Cyanobacteria
c
AJ431236.1.1514 East Pacific Rise hydrothermal vent enrichment clone BHI60-57B
AJ431251.1.1522 East Pacific Rise hydrothermal vent enrichment clone BHI60-1
Zhurongbacter thermophilus 3DAC rRNA 3
Zhurongbacter thermophilus 3DAC rRNA 2
Zhurongbacter thermophilus 3DAC rRNA 1
AJ431252.1.1521 East Pacific Rise hydrothermal vent enrichment clone BHI60-2
AJ431258.1.1520 East Pacific Rise hydrothermal vent enrichment clone Dex80-3
AJ431255.1.1521 East Pacific Rise hydrothermal vent enrichment clone Dex80-4
FN436028.1.1484 Thermophilic biogas reactor clone HAW-R60-B-609d-G
DQ424927.1.1497 Thermophilic microbial fuel cell enriched with artificial wastewater clone C55
DQ424921.1.1497 Thermophilic microbial fuel cell enriched with artificial wastewater clone D84
FN436194.1.1509 Thermophilic biogas reactor clone HAW-R60-B-1249d-U
CP001145.1523 Coprothermobacter proteolyticus DSM 5265
EU591633.1.1457 Anaerobic methanogenic reactor clone HZNBL-H6
FJ769513.1.1480 Full-scale municipal anaerobic digester clone noFP_E11:3
FJ769503.1.1455 Full-scale municipal anaerobic digester clone noFP_F3:3
JQ088349.1.1481 Crude oil reservoir clone MW-B33
GU120594.1.1461 Southern part of Pitch Lake clone PL-B1_4_3_D01
ARJK01000003.1510 Coprothermobacter platensis DSM 11748
AB332116.1.1462 Methanogenic bioreactor clone: EPE305
EF095430.1.1448 Sweet crude oil clone MOB286
EF095419.1.1537 Sweet crude oil clone MOB178
FJ769497.1.1411 Full-scale municipal anaerobic digester clone noFP_H6:2
FJ769474.1.1464 Full-scale municipal anaerobic digester clone FP_F12
Coprothermobacterota
Tree scale: 0.1
Bootstrap >=0.7

Supplementary Figure 5 | Maximum-likelihood phylogeny of the 16S rRNA gene tree. a, Tree with archaeal sequences. b, Tree without archaeal sequences. Red branches in a and b represent the thermophilic lineages involved in this study. c, Tree with detailed 16S rRNA gene sequence information of Zhurongbacter and Coprothermobacterota. The gray squares indicate a bootstrap higher than 0.7.

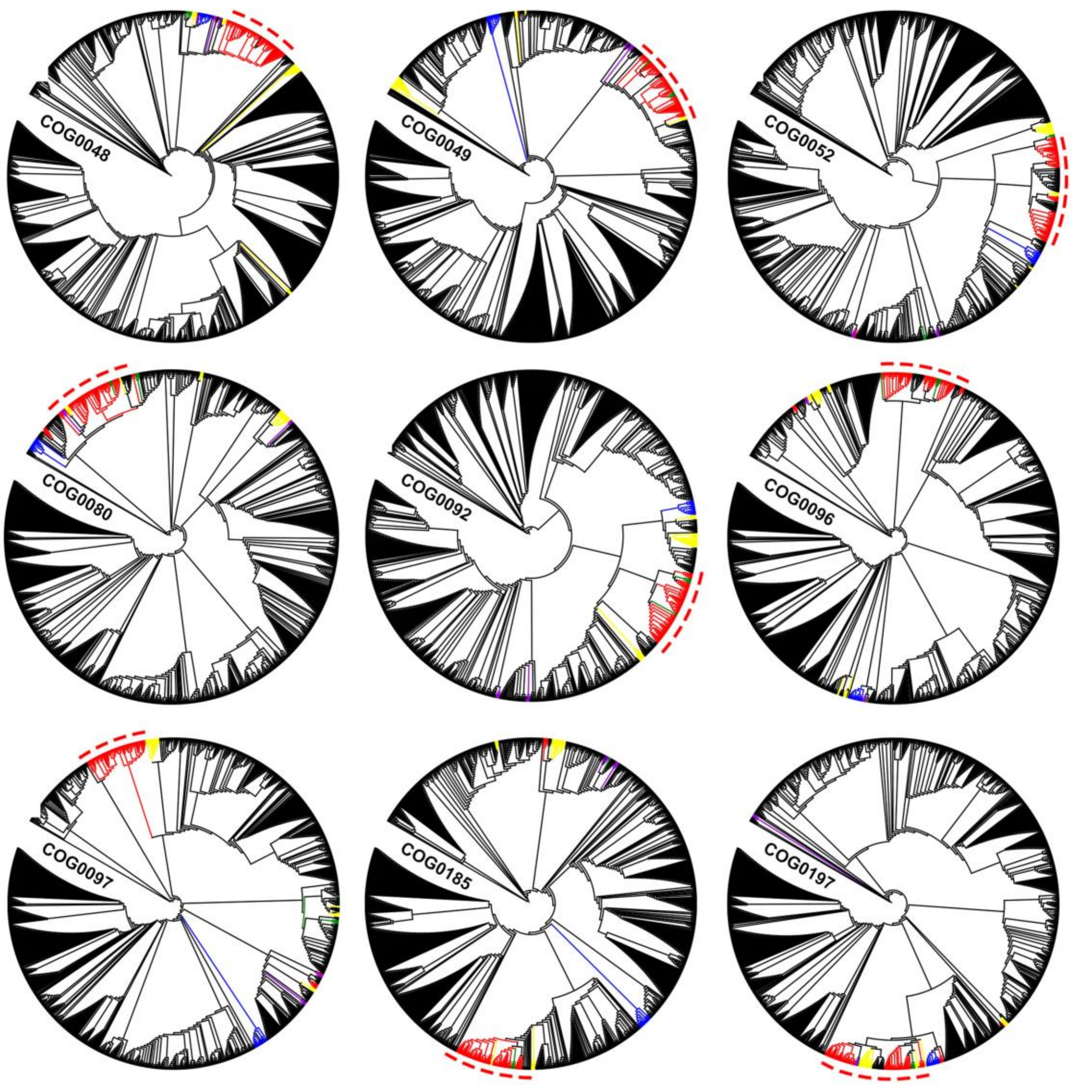

Supplementary Figure 6 | Phylogenetic trees of selected single conserved protein sequences. Red indicates the thermophilic lineages, and Aquificota here is also colored in red. Blue represents Synergistota, while yellow represents Atribacterota and Fusobacteriota. Green indicates Caldisericota.

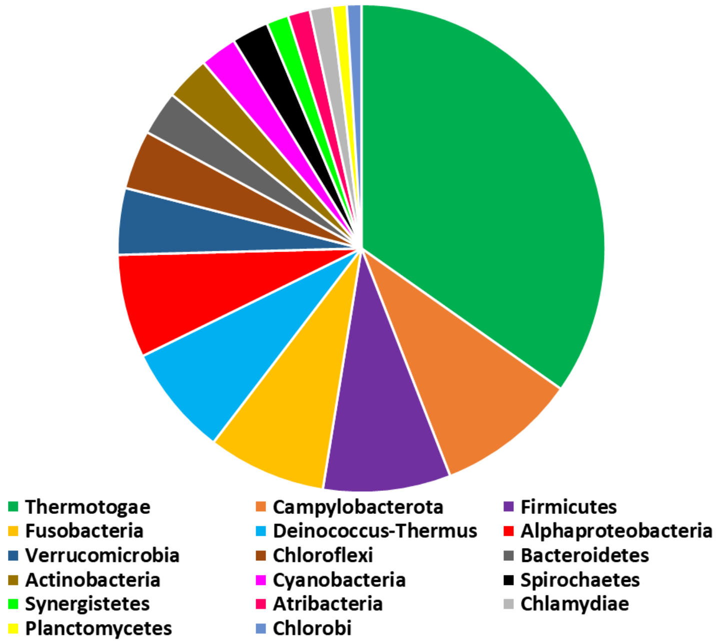

Supplementary Figure 7 | Phylogenetic results for the donor lineages for the HGTs of the conserved proteins. Thirty-seven conserved protein phylogenetic trees were constructed separately to determine whether Aquificota are closely related to the CCTB.

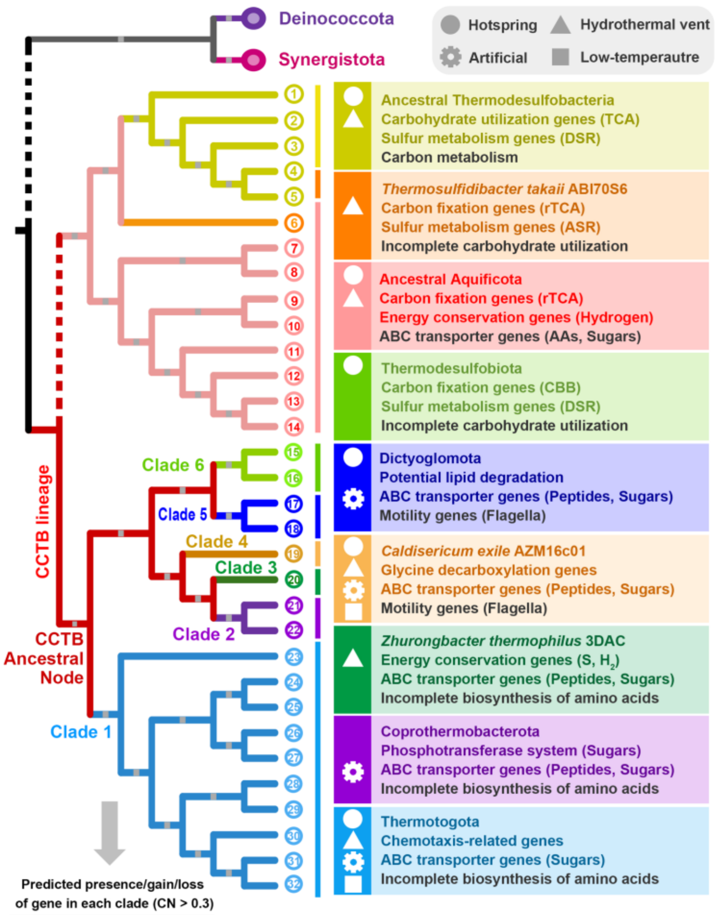

	P	G	L
A. Node	851	/	/
Clade 1	1115	325	61
Clade 2	960	409	300
Clade 3	906	389	336
Clade 4	949	362	266
Clade 5	1145	522	230
Clade 6	1103	554	302

Supplementary Figure 8 | Evolutionary adaptation of the thermophilic lineages. Different colors represent different phyla. Yellow: Thermodesulfobacteriota; Orange: Thermosulfidibacter; Pink: Aquificota; Grass green: Thermodesulfobiota; Blue: Dictyoglomota; Brown: Caldisericota; Dark green: Zhurongbacter; Purple: Coprothermobacterota; Cyan: Thermotogota. The numbers in the node of CCTB are the predicted ancestor gene with a potential gene copy number larger than 0.3 with the presence and absence of genes on major nodes. The complete genomes used in the current study are listed in Supplementary Data 9.

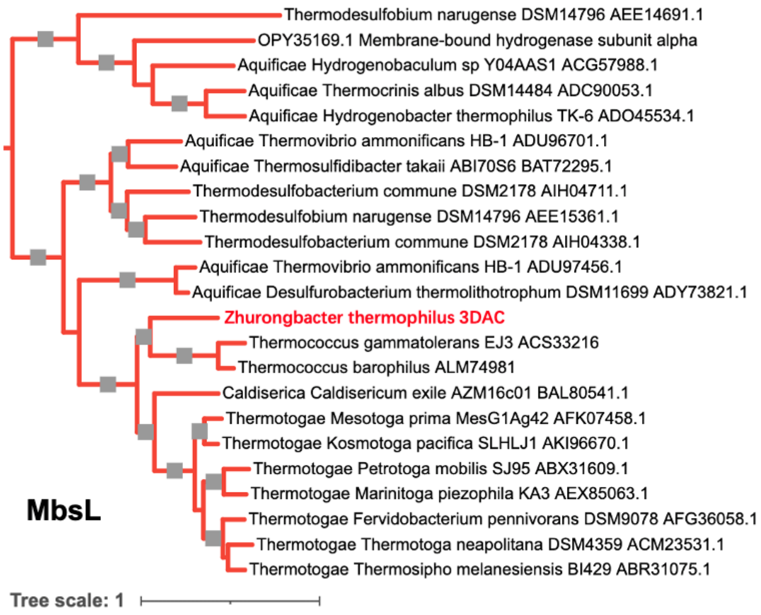

Supplementary Figure 9 | Phylogenetic tree of MbsL protein sequences from the thermophilic lineages. The gray squares indicate a bootstrap higher than 0.8.

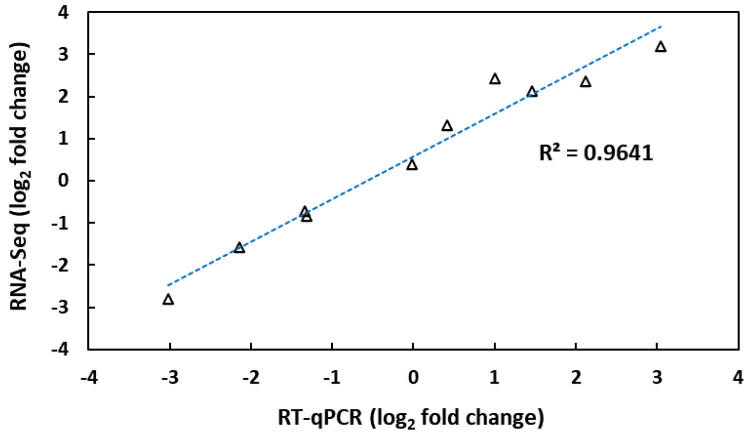

Supplementary Figure 10 | Correlation analysis of the RNA-Seq data and RT-qPCR assays. The horizontal x-axis indicates the log2-fold change according to the RT-qPCR results, the vertical y-axis represents the log2-fold change according to the RNA-Seq results, and R2 indicates the R-square of the regression line.
