# Supplementary material for: Identification of a deep-branching thermophilic clade sheds light on early bacterial evolution
Source: Nat Commun. 2023 Jul 19;14:4354. doi: 10.1038/s41467-023-39960-x (PMC10356935; doi:10.1038/s41467-023-39960-x)
Supplement: Supplementary file 2 — Description of Additional Supplementary Files [file 41467_2023_39960_MOESM2_ESM.pdf]

## Description of Additional Supplementary Files:

**Supplementary Data 1.** Genome-scale metabolic model of Zhurongbacter thermophilus 3DAC (GEM-i3DAC).

**Supplementary Data 2.** Genome-scale metabolic model of the last common ancestor of the CCTB.

**Supplementary Data 3.** AAI values from strain 3DAC to close organisms calculated by compareM.

**Supplementary Data 4.** 16S rRNA gene sequence identities from query sequences to top ten organisms hits by BLASTN.

**Supplementary Data 5.** 16S rRNA gene sequence identities from Zhurongbacterota sequences to Coprothermobacterota sequences (>1400bp) by BLASTN.

**Supplementary Data 6.** Differential characteristics among the members of the CCTB.

**Supplementary Data 7.** Primers used for RT-qPCR.

**Supplementary Data 8.** The 16 conserved protein sequences information.

**Supplementary Data 9.** 37 conserved protein sequence information.

**Supplementary Data 10.** 62 conserved protein sequence information.

**Supplementary Data 11.** Complete genomes used in the ancestral reconstruction.
