# Supplementary material for: Identification of a deep-branching thermophilic clade sheds light on early bacterial evolution
Source: Nat Commun. 2023 Jul 19;14:4354. doi: 10.1038/s41467-023-39960-x (PMC10356935; doi:10.1038/s41467-023-39960-x)
Supplement: Supplementary file 6 — Reporting Summary [file 41467_2023_39960_MOESM6_ESM.pdf]

nature portfolio

Corresponding author(s): Xiang Xiao

Last updated by author(s): Jun 9, 2023

Reporting Summary

Nature Portfolio wishes to improve the reproducibility of the work that we publish. This form provides structure for consistency and transparency in reporting. For further information on Nature Portfolio policies, see our Editorial Policies and the Editorial Policy Checklist.

Statistics

For all statistical analyses, confirm that the following items are present in the figure legend, table legend, main text, or Methods section.

n/a	Confirmed	
☐	☒	The exact sample size (*n*) for each experimental group/condition, given as a discrete number and unit of measurement
☐	☒	A statement on whether measurements were taken from distinct samples or whether the same sample was measured repeatedly
☐	☒	The statistical test(s) used AND whether they are one- or two-sided<br>*Only common tests should be described solely by name; describe more complex techniques in the Methods section.*
☒	☐	A description of all covariates tested
☒	☐	A description of any assumptions or corrections, such as tests of normality and adjustment for multiple comparisons
☐	☒	A full description of the statistical parameters including central tendency (e.g. means) or other basic estimates (e.g. regression coefficient) AND variation (e.g. standard deviation) or associated estimates of uncertainty (e.g. confidence intervals)
☐	☒	For null hypothesis testing, the test statistic (e.g. *F*, *t*, *r*) with confidence intervals, effect sizes, degrees of freedom and *P* value noted<br>*Give P values as exact values whenever suitable.*
☒	☐	For Bayesian analysis, information on the choice of priors and Markov chain Monte Carlo settings
☒	☐	For hierarchical and complex designs, identification of the appropriate level for tests and full reporting of outcomes
☒	☐	Estimates of effect sizes (e.g. Cohen's *d*, Pearson's *r*), indicating how they were calculated

Our web collection on statistics for biologists contains articles on many of the points above.

Software and code

Policy information about availability of computer code

Data collection	Nikon Eclipse 90i fluorescence microscope, FEI Tecnai G2 Spirit Biotwin transmission electron microscope, HACH DR5000 spectrophotometer, PacBio RSII SMRT sequencing platform, Illumina HiSeq X Ten sequencing platform, Illumina MiSeq sequencing platform, Applied Biosystems StepOnePlus Real-Time PCR instrument
Data analysis	Falcon v0.3.0, Celera Assembler v8.3, GATK v1.6-13, Glimmer v3.02, Prodigal v2.6.3, RNAmmer v1.2, tRNAscan-SE v1.3.1, CompareM v.0.0.23, BlastKOALA v2.2, emapper-2.0.1b-2-g816e190, QIIME version 1.9.1, FastQC, Trimmomatic, sortmerna, Bowtie 2, DESeq2 package, Primer-BLAST, MAFFT v7.313, SINA v1.2.11, IQ-TREE v2.0.6, trimAl v1.4.rev2, iTOL online software, amalgamated likelihood estimation (ALE) version 0.4, PSAMM version 1.1.1, IBM ILOG CPLEX Optimizer version 12.7.1.0 , eggNOG-Mapper v2.0.1b-2-g816e190, OrthoFinder v2.5.4

For manuscripts utilizing custom algorithms or software that are central to the research but not yet described in published literature, software must be made available to editors and reviewers. We strongly encourage code deposition in a community repository (e.g. GitHub). See the Nature Portfolio guidelines for submitting code & software for further information.

Data

Policy information about availability of data

All manuscripts must include a data availability statement. This statement should provide the following information, where applicable:

- Accession codes, unique identifiers, or web links for publicly available datasets
- A description of any restrictions on data availability
- For clinical datasets or third party data, please ensure that the statement adheres to our policy

All data are stored in NCBI database. The genome sequence of Zhurongbacter thermophilus 3DAC was reconstructed and deposited under accession number CP046447. Amplicon sequencing data have been deposited at SRA under accession numbers SRR14072822, SRR14072823, SRR14072817, SRR14072825,

SRR14072824, SRR14072798, and SRR14072797, and transcriptome sequence data have been deposited at SRA under accession numbers SRR14072890 and SRR14072891.

Field-specific reporting

Please select the one below that is the best fit for your research. If you are not sure, read the appropriate sections before making your selection.

- [x] Life sciences
- [ ] Behavioural & social sciences
- [ ] Ecological, evolutionary & environmental sciences

For a reference copy of the document with all sections, see nature.com/documents/nr-reporting-summary-flat.pdf

Life sciences study design

All studies must disclose on these points even when the disclosure is negative.

Sample size	Growth experiments were performed in three biological replicates. The substrate utilization experiments were performed in duplicates. RNA-seq analysis was performed in three independent cultivation experiments based on statistical requirements.
Data exclusions	No data were excluded from analysis.
Replication	Replication was performed as described in all the experiments reported. Physiological tests have biological replicates, quantitative PCR not only has biological replicates, but also technical replicates. All attempts at replication were successful.
Randomization	The tests of substrate utilization have a certain randomness, and we only tested some common substrates.
Blinding	Blinding was not applicable because this study was designed to collect the physiological data of the isolated thermophile.

Reporting for specific materials, systems and methods

We require information from authors about some types of materials, experimental systems and methods used in many studies. Here, indicate whether each material, system or method listed is relevant to your study. If you are not sure if a list item applies to your research, read the appropriate section before selecting a response.

Materials & experimental systems

n/a	Involved in the study	
x		Antibodies
x		Eukaryotic cell lines
x		Palaeontology and archaeology
x		Animals and other organisms
x		Human research participants
x		Clinical data
x		Dual use research of concern

Methods

n/a	Involved in the study	
x		ChIP-seq
x		Flow cytometry
x		MRI-based neuroimaging
